# Navigation-Assisted Minimally Invasive Transforaminal Lumbar Interbody Fusion (MIS-TLIF) for Lumbar Facet Synovial Cysts Associated With Degenerative Spondylolisthesis: A Case Series and Surgical Technique

**DOI:** 10.7759/cureus.112003

**Published:** 2026-07-03

**Authors:** Armando Francisco Perez Castell, Jose I Fernández-Faudoa, Alfredo Porras Mendoza, Carlos A Arellanes-Chavez

**Affiliations:** 1 Neurosurgery/Spine Surgery, Star Medica/Universidad Autonoma de Chihuahua, Chihuahua, MEX; 2 Neurosurgery, Star Médica, Chihuahua, MEX; 3 Neurosurgery, Star Médica/Centro Innovador de Servicios Neuroquirúrgicos (CISNE) Spine, Chihuahua, MEX; 4 Microneurosurgery/Neurosurgery/Spine Surgery, Star Médica/Centro Innovador de Servicios Neuroquirúrgicos (CISNE) Spine, Chihuahua, MEX

**Keywords:** lumbar facet synovial cyst, lumbar spondylolisthesis, minimally invasive spine surgery, neuronavigation, spine neuronavigation

## Abstract

Lumbar facet synovial cysts (LFSCs) are degenerative lesions associated with facet arthropathy and segmental instability, predominantly occurring at the L4-L5 level. They may cause radiculopathy and neurogenic claudication, and although conservative management may provide temporary relief, cases associated with degenerative spondylolisthesis (DS) frequently require surgical treatment. Minimally invasive spine surgery assisted by spinal navigation allows precise cyst resection and adequate stabilization. The aim of this study was to present a case series of LFSCs associated with DS treated with navigation-assisted minimally invasive transforaminal lumbar interbody fusion and to describe the surgical technique and role of spinal neuronavigation in minimally invasive decompression and instrumentation. We report a series of three patients with symptomatic LFSCs associated with DS refractory to conservative treatment. All patients were evaluated using magnetic resonance imaging and dynamic radiographs and were treated with cyst resection, neural decompression, and percutaneous instrumentation assisted by neuronavigation. All patients demonstrated significant clinical improvement following surgical treatment, with reduction of radicular pain and functional improvement during postoperative follow-up. Most cases were associated with DS and advanced facet arthropathy changes. Neuronavigation allowed precise cyst resection with adequate neural decompression and preservation of posterior bony structures, without major complications or early recurrence during follow-up. LFSCs are closely associated with lumbar degenerative instability, and navigation-assisted resection represents a safe and effective minimally invasive alternative allowing adequate decompression while preserving spinal anatomy. Modern minimally invasive techniques and interventional therapies are emerging as complementary options in selected patients.

## Introduction

Lumbar facet synovial cysts (LFSCs) are uncommon degenerative lesions originating from the capsule of the zygapophyseal joints and may cause low back pain, radiculopathy, and neurogenic claudication due to neural compression [1]. In magnetic resonance imaging (MRI) studies performed for low back or radicular pain, an approximate prevalence of 9.6% has been reported, with 2.3% protruding into the spinal canal [2]. These lesions show an estimated incidence ranging from 0.5% to 2.3%, with a predominance at the L4-L5 level [3].

LFSCs develop as a consequence of degenerative facet joint processes and are closely associated with segmental instability and degenerative spondylolisthesis (DS) [1,4-6]. Most lesions occur at the L4-L5 level, the most mobile segment of the lumbar spine [7]. From a pathophysiological perspective, intervertebral disc degeneration and increased posterior loading elevate stress on the facet joints, promoting synovitis, excessive fluid production, and eventual cyst formation [3,6,8]. The concomitant presence of DS has been reported in up to 38-50% of cases, reinforcing the hypothesis that instability plays a central role in the development of these lesions [9,10].

DS is a common condition in older adults, particularly at the L4-L5 level, and is associated with progressive facet degeneration and segmental instability [3]. Throughout its natural history, radiological markers such as facet effusion, facet vacuum phenomenon, and synovial cysts have been described, reflecting different stages of instability and degenerative progression [3]. Structural changes of the facet joints, including erosions, sclerosis, ankylosis, and osseous remodeling, correlate with segmental biomechanical alterations and may contribute to instability and secondary pathology, including facet cyst formation [11].

MRI is the diagnostic modality of choice because it allows identification of the cyst, its relationship to adjacent nerve roots, and indirect signs of instability such as facet effusion. The presence of facet fluid greater than 1 mm has been significantly correlated with dynamic instability and DS [8,12].

Several radiological classifications have been proposed to guide therapeutic decision-making and estimate recurrence risk, including the NeuroSpine Surgery Research Group (NSURG) grading system and classifications based on cyst location [4,7]. These systems integrate the degree of neural compression and vertebral instability, facilitating surgical planning and helping identify patients who may benefit from concomitant fusion procedures, particularly those with associated DS [7].

Conservative treatment, including rest, physical therapy, and image-guided injections, may provide symptomatic relief in selected cases; however, long-term success rates remain limited, and many patients ultimately require surgical treatment [13,14]. Although isolated reports of spontaneous regression have been described, these remain uncommon and do not represent the standard clinical course [15].

Traditional surgical treatment consists of laminectomy and cyst resection, with or without instrumented fusion. Several studies have reported favorable clinical outcomes following decompression alone; however, there remains a risk of postoperative progression of spondylolisthesis or cyst recurrence, particularly in the presence of preexisting instability [10]. Other authors suggest that laminectomy combined with instrumented posterolateral fusion should be considered the preferred treatment option in symptomatic cases, as it allows adequate neural decompression while reducing recurrence risk, especially in patients with associated instability.

In this context, minimally invasive surgical techniques and spinal navigation systems represent an evolution in the management of this pathology, allowing precise cyst resection, structural preservation, and accurate instrumentation placement in patients with DS. Recent literature supports selective facet resection and surgical strategies tailored to the degree of instability [5,9]. Nevertheless, evidence regarding the application of navigation-assisted minimally invasive transforaminal lumbar interbody fusion (MIS-TLIF) specifically for LFSCs associated with DS remains limited.

Therefore, the aim of this study is to present a case series describing the surgical management of LFSCs associated with DS using navigation-assisted MIS-TLIF, while detailing the surgical technique and highlighting the role of spinal neuronavigation in achieving precise decompression and stable minimally invasive instrumentation.

## Case presentation

Three patients with symptomatic LFSCs associated with degenerative lumbar spondylolisthesis refractory to conservative management were included in this case series. Preoperative evaluation consisted of lumbosacral MRI and dynamic flexion-extension radiographs to confirm the diagnosis and assess segmental instability, while traumatic, infectious, and neoplastic etiologies were excluded. All patients underwent navigation-assisted MIS-TLIF and were evaluated postoperatively at one month and 12 months using the visual analog scale (VAS) and the Oswestry Disability Index (ODI), together with radiographic assessment of fusion status.

Case 1

A 50-year-old female homemaker with a body mass index (BMI) of 30 kg/m² presented with chronic low back pain associated with paresthesia, dysesthesia, weakness, and claudication of the left lower extremity. Neurological examination revealed muscle strength of 4/5 in the left lower extremity, preserved deep tendon reflexes, and intact sensation. Lasègue and Bragard maneuvers were positive on the left side.

Dynamic lumbosacral radiographs demonstrated unstable grade I DS at L4-L5 (Figure 1). Lumbar MRI revealed lumbar canal narrowing and foraminal compromise at L4-L5 secondary to an LFSC causing compression of the exiting nerve roots and compression of the adjacent neural structures (Figure 2).

**Figure 1:**
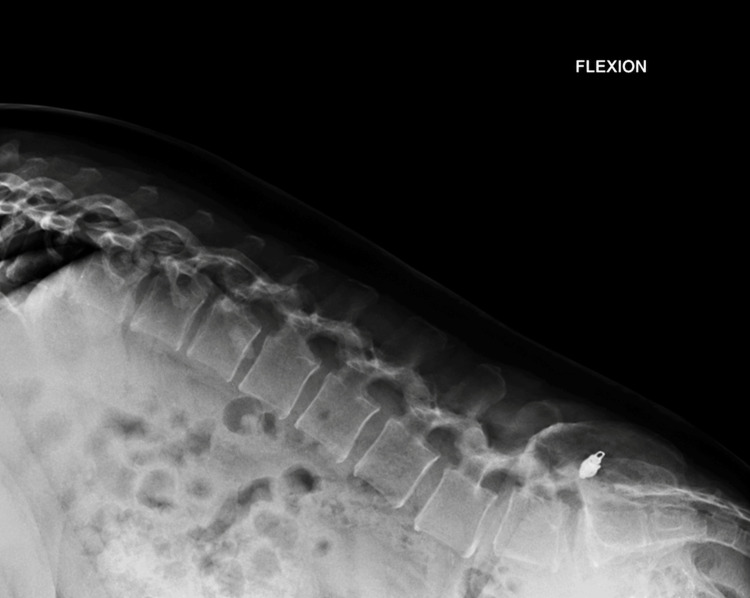
Dynamic flexion radiograph demonstrating L4-L5 spondylolisthesis.

**Figure 2:**
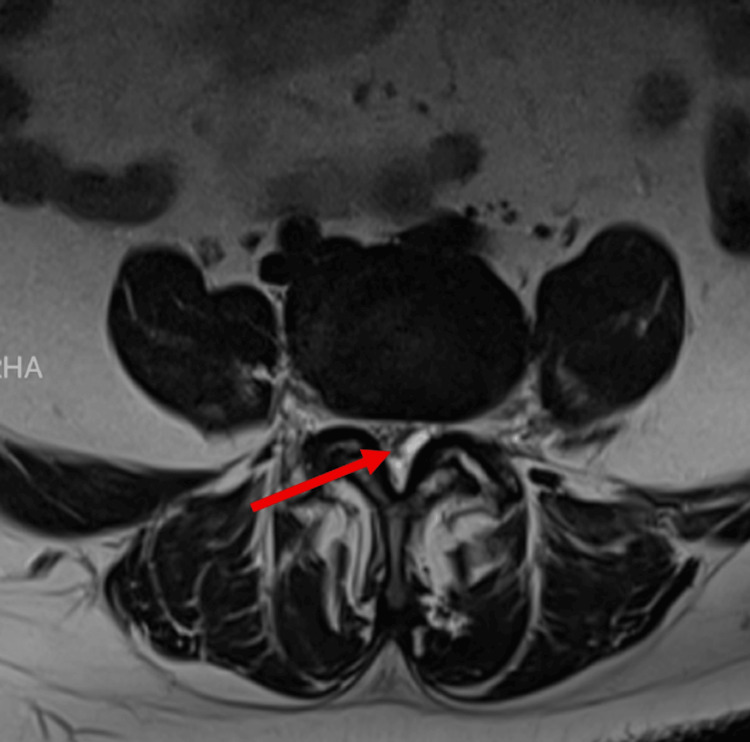
Axial lumbosacral MRI demonstrating a left-sided L4-L5 facet synovial cyst (red arrow) causing compression of the adjacent neural structures.

The patient underwent left-sided MIS-TLIF at L4-L5 with percutaneous pedicle screw placement assisted by neuronavigation using the O-arm system (Medtronic©), synovial cyst resection, and placement of an interbody cage (Figure 3).

**Figure 3:**
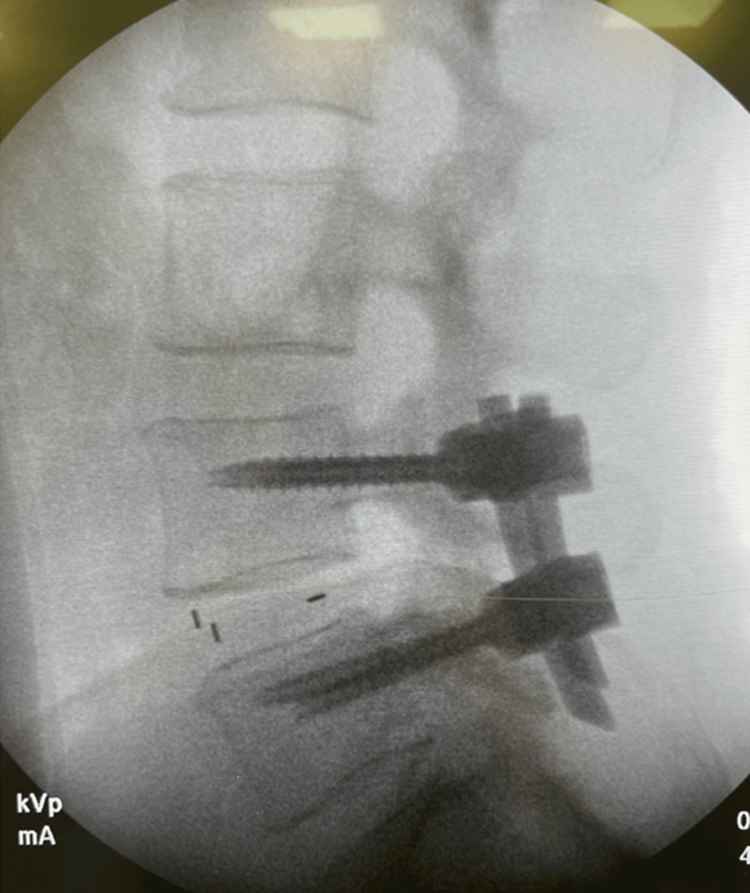
Postoperative radiographic control following MIS-TLIF at L4-L5. MIS-TLIF: Minimally invasive transforaminal lumbar interbody fusion

At one-month postoperative follow-up, the patient demonstrated significant clinical improvement, including reduction of low back and radicular pain, improved mobility, and partial resolution of paresthesias. The ODI improved from 60% preoperatively to 18% postoperatively, while the VAS score improved from 8 to 2 points. At the 12-month follow-up, the patient reported complete resolution of pain (VAS 0/10), required no pain medication, had returned to normal daily activities, and demonstrated an ODI of 3%, reflecting excellent functional recovery.

Case 2

A 55-year-old female merchant with no significant chronic degenerative medical history presented with chronic low back pain since 2023, progressively associated with weakness and paresthesias in the left lower extremity. She had previously undergone conservative and interventional management, including rhizolysis, foraminal block, and nucleoplasty, without significant clinical improvement.

Physical examination revealed persistent radiating low back pain accompanied by dysesthesia and muscle weakness in the left L4-L5 distribution, with muscle strength graded as 4/5. The Lasègue maneuver was positive in the left lower extremity, and the Achilles tendon reflex was decreased (+/++).

Dynamic flexion radiographs demonstrated grade I DS at L4-L5 (Figure 4). Lumbosacral MRI revealed a left-sided L4-L5 facet synovial cyst associated with facet arthropathy, spinal canal narrowing, neural compression, and facet joint effusion (Figure 5).

**Figure 4:**
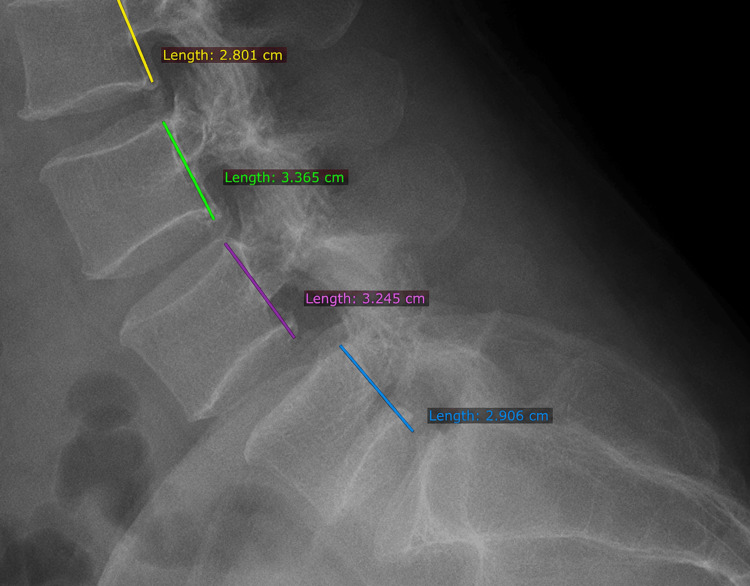
Dynamic flexion radiograph demonstrating low-grade (Meyerding grade I) L4-L5 degenerative spondylolisthesis associated with degenerative sclerosis and segmental instability.

**Figure 5:**
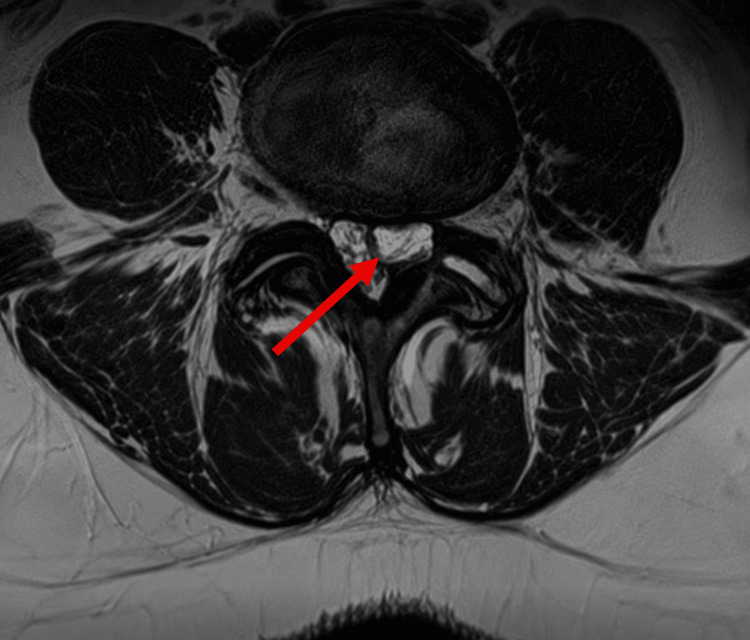
Lumbar magnetic resonance imaging (MRI) demonstrating a left-sided L4-L5 facet synovial cyst (red arrow) associated with facet hypertrophy, facet joint effusion (hydroarthrosis), spinal canal narrowing, and neural compression.

The patient underwent surgical treatment with MIS-TLIF at L4-L5 assisted by neuronavigation, combined with excision of the facet synovial cyst. Postoperative radiographic evaluation demonstrated adequate instrumentation placement (Figure 6).

**Figure 6:**
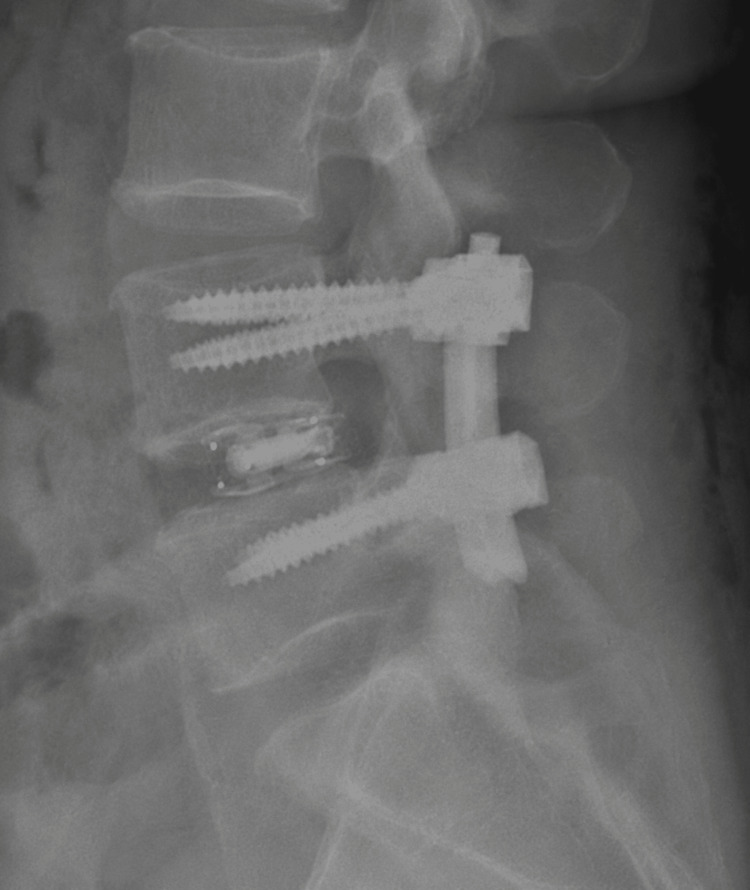
Postoperative radiographic control following MIS-TLIF at L4-L5 demonstrating adequate instrumentation placement and positioning of an expandable interbody cage. MIS-TLIF: Minimally invasive transforaminal lumbar interbody fusion

Histopathological analysis confirmed the diagnosis of an LFSC, demonstrating synovial lining proliferation with reactive synoviocytes and a characteristic fibrous wall (Figure 7).

**Figure 7:**
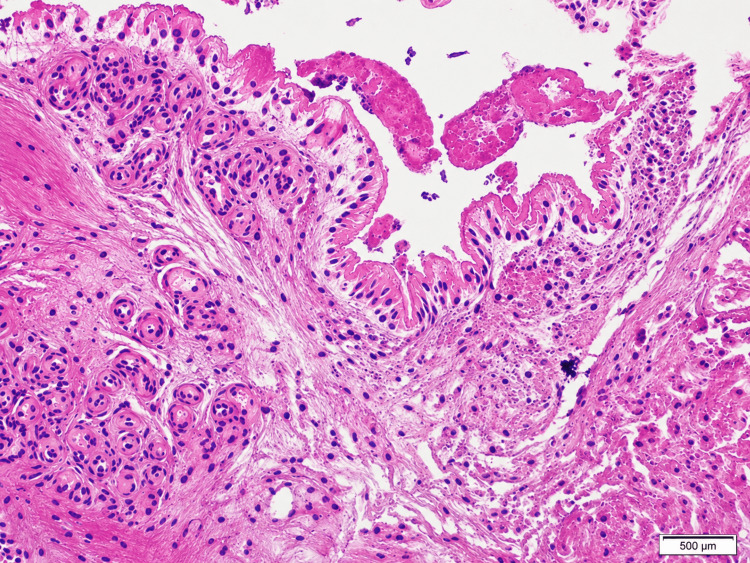
Photomicrograph demonstrating synovial lining proliferation with reactive synoviocytes and a fibrous wall characteristic of a lumbar facet synovial cyst.

At one-month postoperative follow-up, the patient demonstrated significant improvement in pain and mobility. The ODI improved from 70% preoperatively to 25% postoperatively, while the VAS score improved from 9 preoperatively to 4 postoperatively (VAS 0-10). At the 12-month follow-up, the patient showed sustained clinical improvement, with a VAS score of 3/10 and an ODI of 10%. Radiological evaluation confirmed solid fusion, with no evidence of implant loosening or segmental instability.

Case 3

A 61-year-old male with grade I obesity and arterial hypertension presented with chronic left-sided lumbosciatic pain associated with paresthesias and dysesthesias. Relevant medical history included two prior lumbar endoscopic procedures performed since 2022, as well as pharmacological treatment with pregabalin, without significant clinical improvement.

Neurological examination revealed preserved muscle strength and sensation, bilateral Achilles deep tendon reflexes graded as ++/++, and a positive Lasègue maneuver in the left lower extremity.

Dynamic lumbosacral radiographs demonstrated DS at L4-L5 Grade 1 Meyerding (Figure 8). Lumbar MRI revealed a left-sided L4-L5 facet synovial cyst associated with neural compression, grade 2 fatty infiltration, facet hypertrophy, and facet joint effusion (Figure 9).

**Figure 8:**
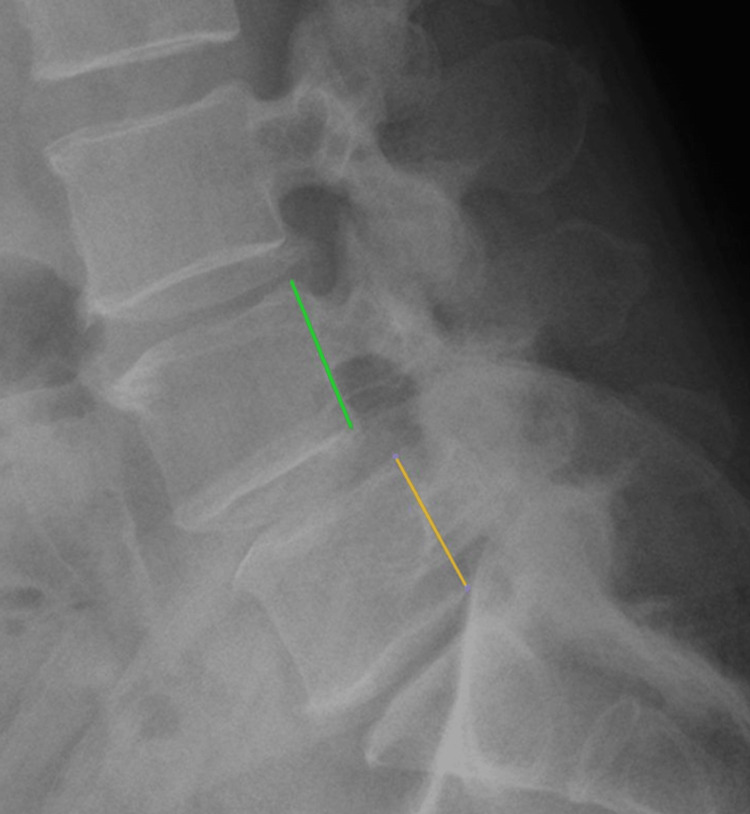
Dynamic lateral lumbar radiograph demonstrating grade I degenerative spondylolisthesis at L4-L5 according to the Meyerding classification.

**Figure 9:**
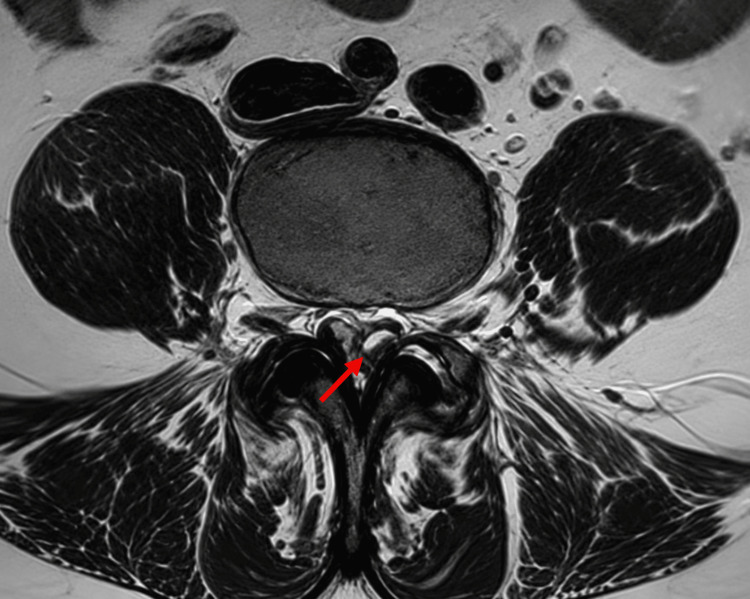
Axial lumbar magnetic resonance imaging (MRI) demonstrating a left-sided L4-L5 facet synovial cyst (red arrow) associated with grade 2 fatty infiltration, facet hypertrophy, and facet joint effusion.

The patient underwent surgical treatment with MIS-TLIF at L4-L5 assisted by neuronavigation, combined with excision of the facet synovial cyst. Postoperative radiographic evaluation demonstrated adequate instrumentation placement (Figure 10).

**Figure 10:**
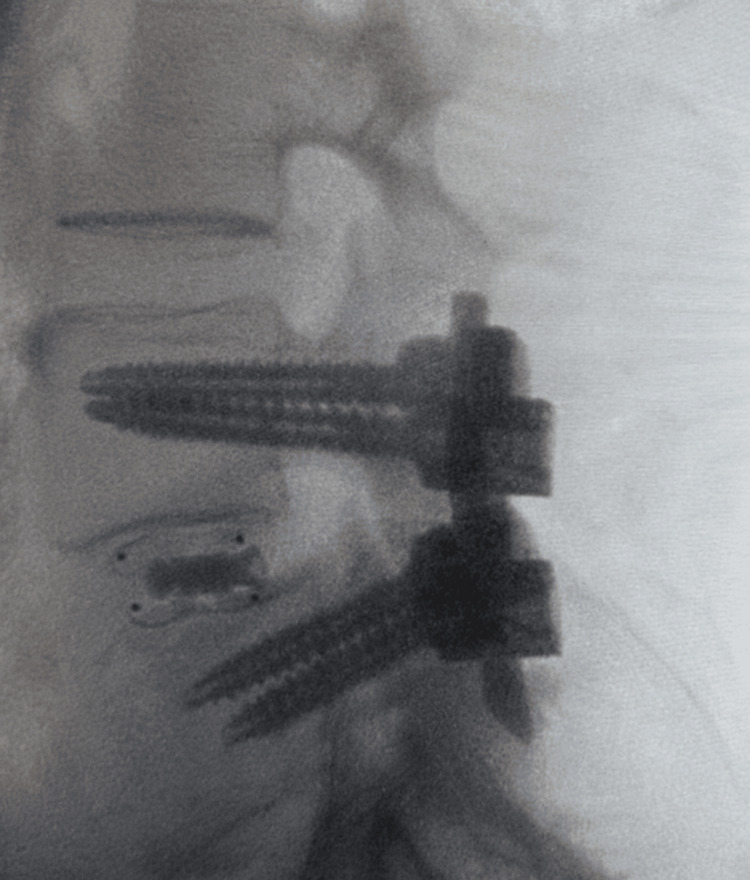
Postoperative radiographic control following MIS-TLIF at L4-L5 demonstrating adequate pedicle screw instrumentation and placement of an expandable interbody cage with restoration of intervertebral disc height. MIS-TLIF: Minimally invasive transforaminal lumbar interbody fusion

Histopathological examination confirmed the diagnosis of an LFSC (Figure 11).

**Figure 11:**
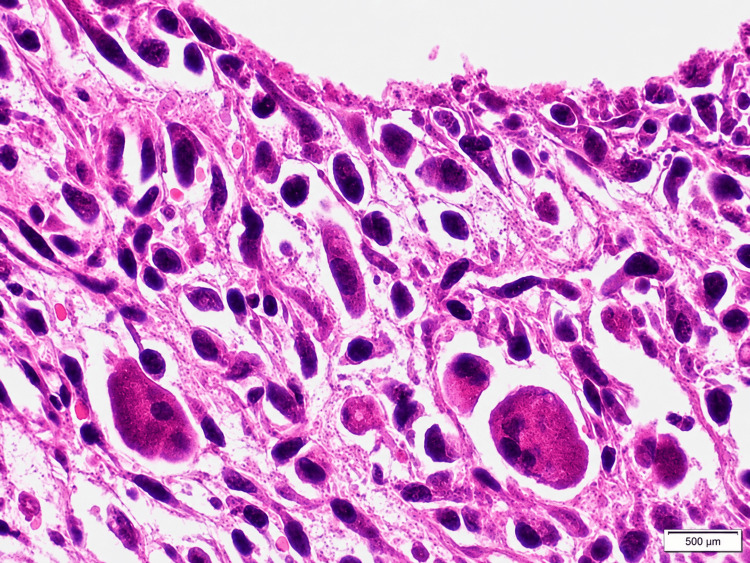
High-power photomicrograph demonstrating synovial lining with reactive synoviocyte proliferation associated with granulomatous reaction and multinucleated giant cells within a fibrocollagenous stroma. Hematoxylin and eosin stain, 400× magnification.

At one-month postoperative follow-up, the patient demonstrated significant improvement in low back and radicular pain, as well as reduction of paresthesias. The ODI improved from 50% preoperatively to 15% postoperatively, while the VAS score improved from 7 to 2. At the 12-month follow-up, the patient reported complete resolution of pain (VAS 0/10) and achieved an ODI of 0%, indicating full functional recovery and solid arthrodesis, with stable implant positioning and no signs of hardware loosening or recurrent instability (Figure 12).

**Figure 12:**
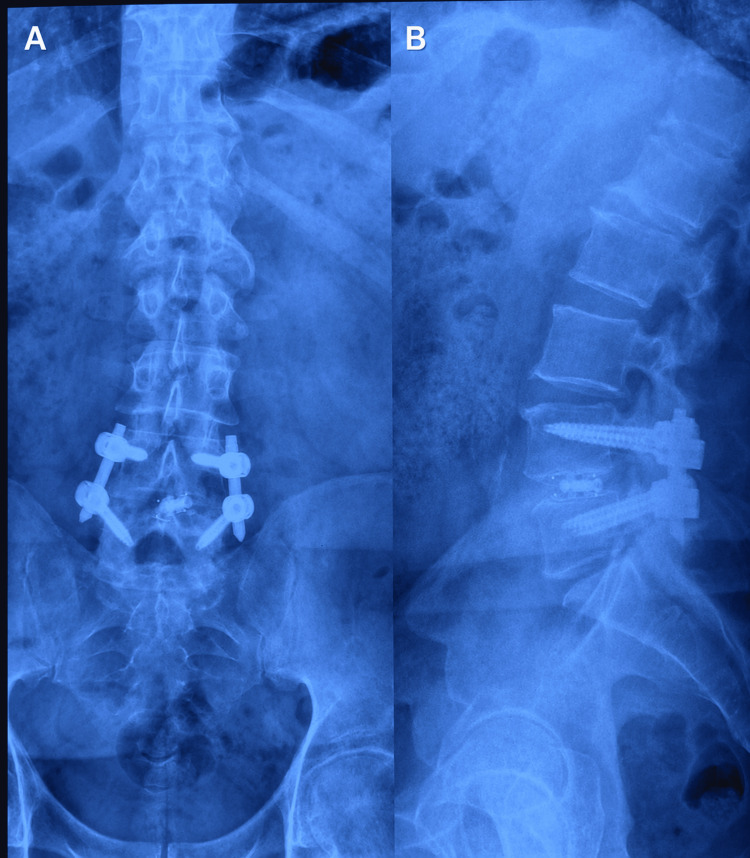
(A) Anteroposterior and (B) lateral radiographs at 12 months postoperatively, demonstrating adequate solid fusion with stable instrumentation and satisfactory alignment.

Navigation-assisted MIS-TLIF technique and facet synovial cyst excision

Step 1. Placement of the Reference System

The patient was positioned prone on a radiolucent table under general anesthesia. Sterile preparation and draping were performed, followed by placement of the reference pin in the right iliac crest for the neuronavigation system (Figure 13).

**Figure 13:**
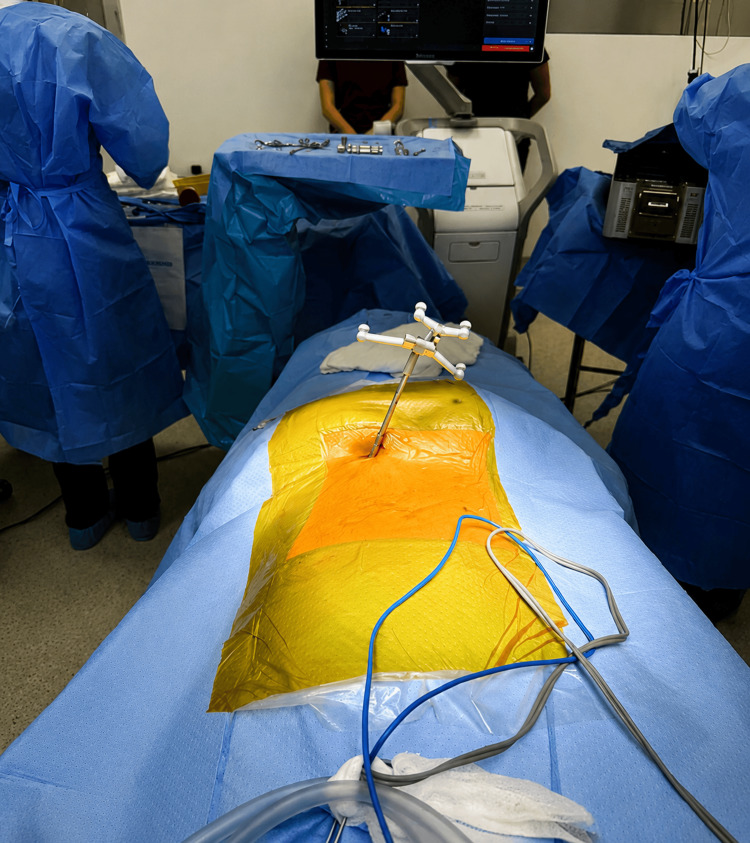
Placement of the reference pin in the right iliac crest.

Step 2. Intraoperative Image Acquisition

An intraoperative O-arm scan was obtained for three-dimensional image acquisition and spinal anatomical registration, allowing real-time navigation throughout the procedure (Figure 14).

**Figure 14:**
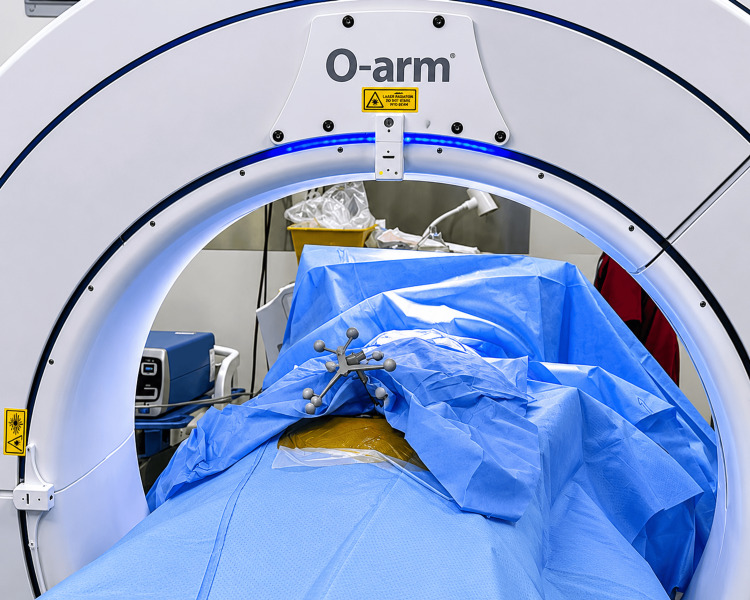
Intraoperative spinal imaging using the O-arm system.

Step 3. Pedicle Trajectory Planning

Initially, navigated planning of the pedicle screw trajectories was performed. Percutaneous Jamshidi needles were inserted under neuronavigation guidance for bilateral pedicle cannulation at L4 and L5 (Figure 15).

**Figure 15:**
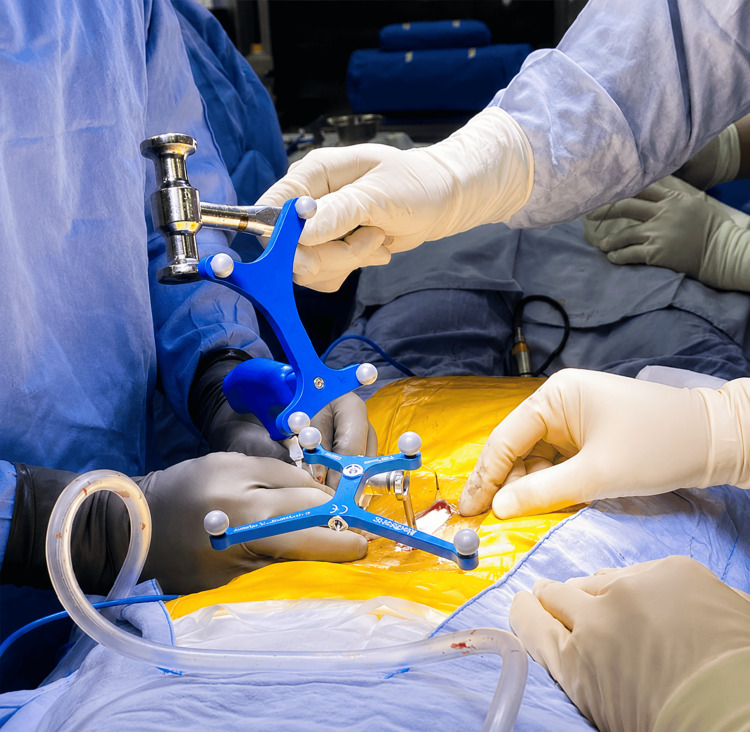
Intraoperative placement of the navigated Jamshidi needle and pedicle reference frame during planning of the pedicle trajectory for percutaneous instrumentation.

Step 4. Navigated Preparation of Pedicle Trajectories

Figure 16 demonstrates the navigated pedicle trajectory used for insertion of the Jamshidi needle, allowing accurate pedicle orientation and facilitating subsequent pedicle screw placement.

**Figure 16:**
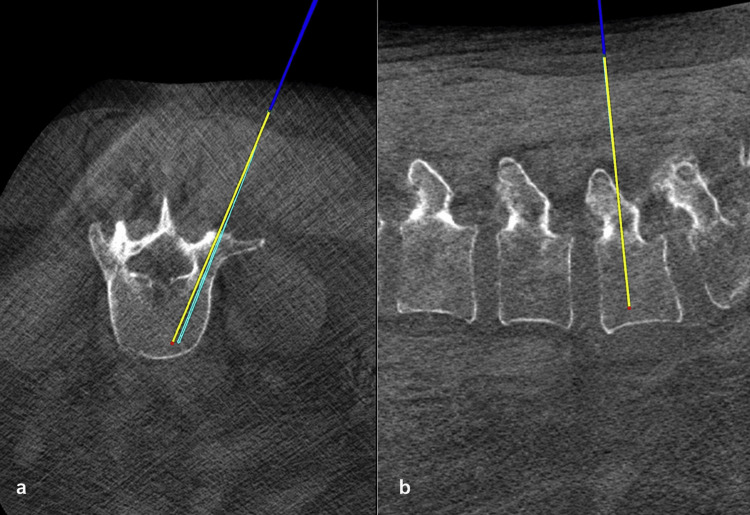
Navigation-assisted Jamshidi needle trajectory. (A) Axial view demonstrating the planned pedicle trajectory. (B) Sagittal view confirming the navigated trajectory.

Step 5. Lateral Fixation of Needles and Guidewires

The needles and guidewires were secured laterally using surgical clamps outside the tubular working corridor, preventing interference during the decompression and facetectomy stages.

Step 6. Tubular Approach and Facetectomy

A minimally invasive tubular approach was then performed with unilateral facetectomy using an expandable tubular retractor system (Figure 17).

**Figure 17:**
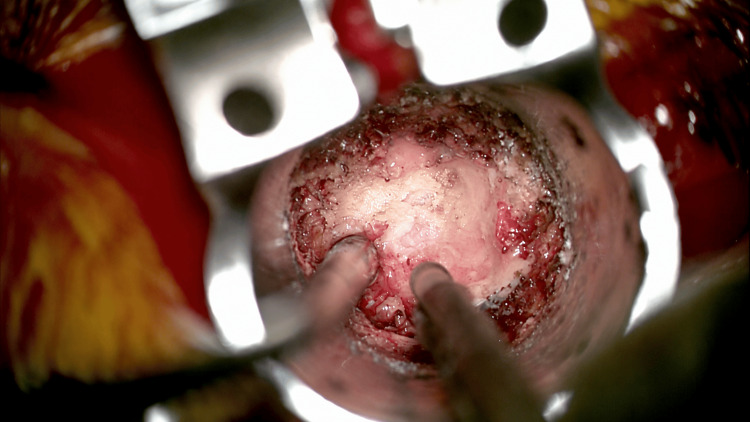
Facetectomy performed using a tubular retractor system.

Step 7. Identification of the Facet Cyst

Neuronavigation was used to accurately identify the anatomical location of the facet synovial cyst and its relationship to adjacent neural structures (Figure 18).

**Figure 18:**
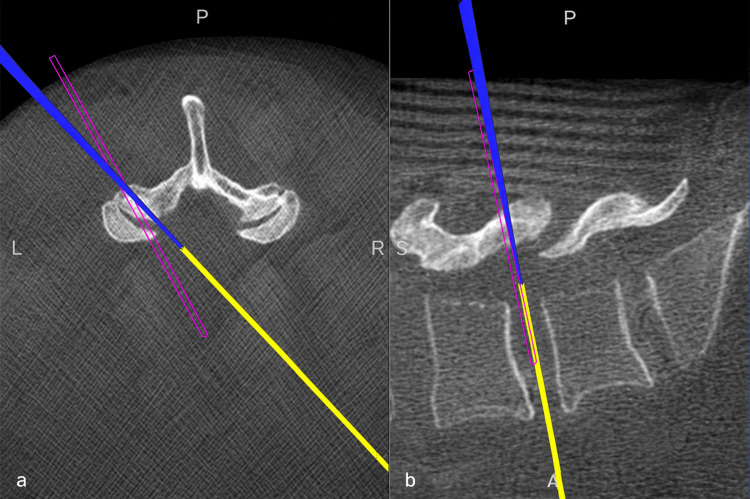
Navigation-assisted localization of the lumbar facet synovial cyst. (A) Axial view showing the planned surgical corridor. (B) Sagittal view confirming the target and surgical trajectory.

Step 8. Microsurgical Cyst Resection

Microsurgical excision of the LFSC was performed using tubular instrumentation and a microscopic technique, achieving adequate neural decompression (Figure 19).

**Figure 19:**
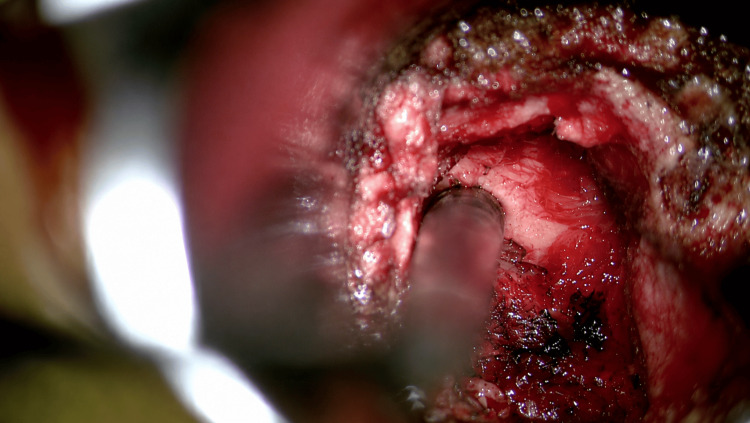
Facet synovial cyst resection using a tubular retractor system.

Step 9. Interbody Cage Placement

After completion of the discectomy and endplate preparation, an interbody cage was inserted under neuronavigation guidance, confirming adequate intraoperative positioning (Figure 20).

**Figure 20:**
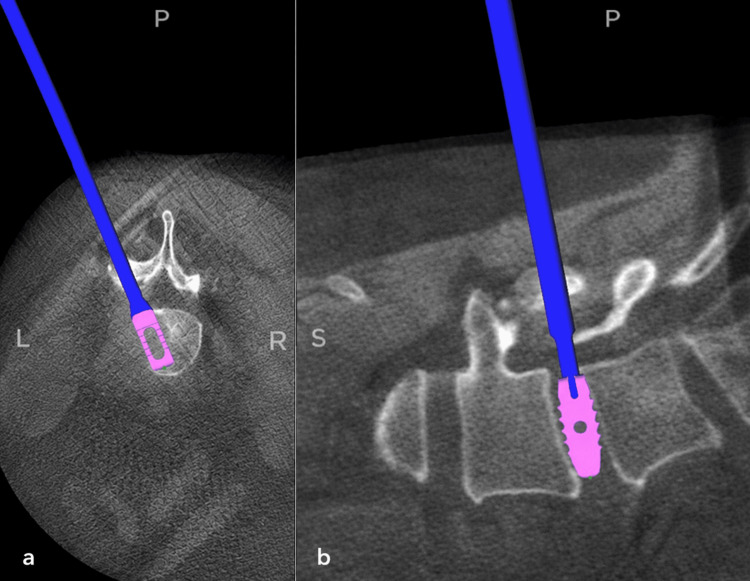
Interbody cage placement assisted by neuronavigation. (A) Axial plane demonstrating the navigated trajectory and positioning of the interbody cage within the disc space. (B) Sagittal plane confirming accurate placement of the interbody cage during minimally invasive transforaminal lumbar interbody fusion (MIS-TLIF).

Step 10. Pedicle Screw Placement

Percutaneous pedicle screws were subsequently placed through the previously planned trajectories using neuronavigation assistance (Figure 21).

**Figure 21:**
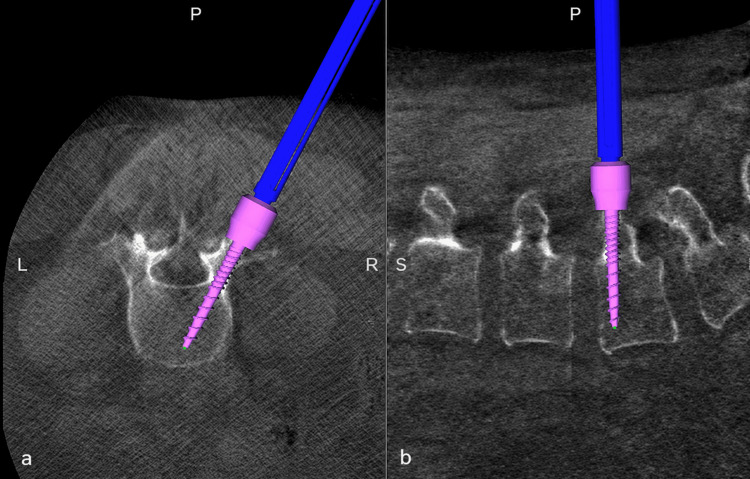
Navigation-assisted percutaneous pedicle screw placement. (A) Axial view showing the navigated pedicle screw trajectory. (B) Sagittal view confirming accurate pedicle screw placement.

Step 11. Rod Placement and Final Control

Finally, percutaneous rod placement was performed under fluoroscopic guidance, obtaining satisfactory final radiographic control with adequate positioning of the interbody cage, pedicle screws, and posterior rods following MIS-TLIF at L4-L5 (Figure 22).

**Figure 22:**
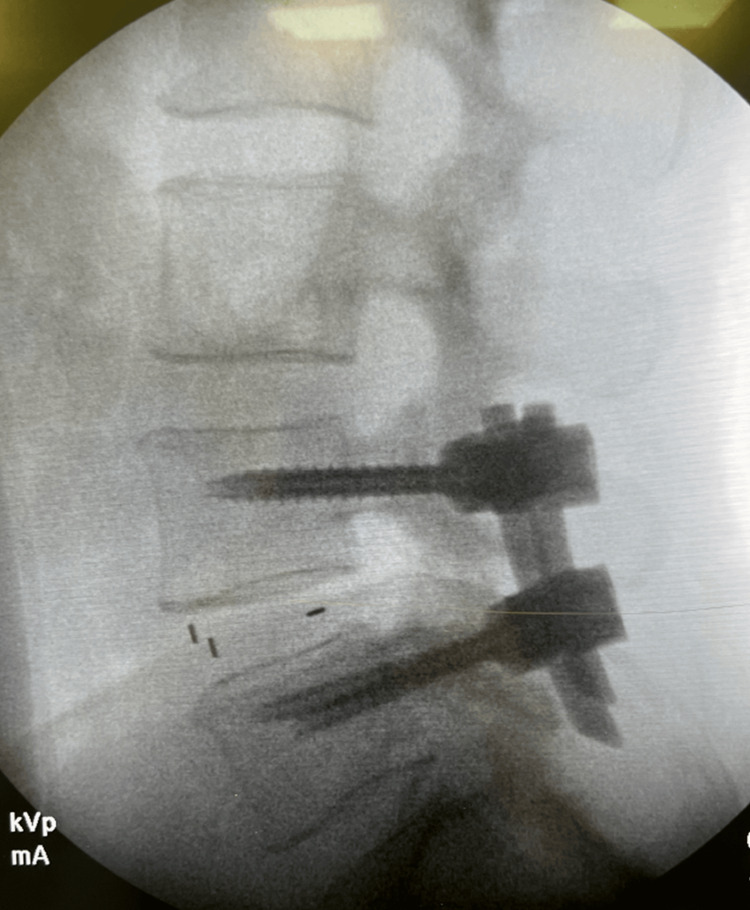
Percutaneous rod placement under fluoroscopic guidance and final postoperative control demonstrating adequate positioning of the interbody cage, pedicle screws, and rods following MIS-TLIF at L4-L5. MIS-TLIF: Minimally invasive transforaminal lumbar interbody fusion

Results

Three patients with symptomatic LFSCs associated with L4-L5 DS refractory to conservative treatment were included. Two patients were female and one was male, with ages ranging from 50 to 61 years. Individual clinical, radiological, surgical, and postoperative characteristics are summarized in Table 1, whereas the aggregated clinical outcomes, surgical variables, and 12-month follow-up results are summarized in Table 2.

**Table 1:** Clinical, surgical, and postoperative outcomes at one- and 12-month follow-up in patients with lumbar facet synovial cysts treated with navigation-assisted MIS-TLIF MIS-TLIF: Minimally invasive transforaminal lumbar interbody fusion; ODI: Oswestry Disability Index; VAS: visual analog scale

Case	Age/Sex	Level	Main Symptoms	Previous Treatment	Procedure	ODI Pre→Post 1 month	VAS Pre→Post 1 month	ODI at 12 months	VAS at 12 months	Operative time (min)	Estimated blood loss (mL)	Hospital stay (days)	Perioperative complications	Fusion status	Outcome
1	50/F	L4-L5	Low back pain, paresthesias, claudication, and left-sided weakness	Conservative treatment	MIS-TLIF + cyst resection + neuronavigation	60% → 18%	8 → 2	3%	0	210	100	3	No	Solid fusion achieved	Significant improvement
2	55/F	L4-L5	Low back pain, dysesthesias, and left-sided weakness	Rhizolysis, foraminal block, and nucleoplasty	MIS-TLIF + cyst resection + neuronavigation	70% → 25%	9 → 4	10%	3	210	50	2	No	Solid fusion achieved	Clinical and functional improvement
3	61/M	L4-L5	Lumbosciatic pain, paresthesias, and dysesthesias	Previous endoscopic surgeries and pregabalin	MIS-TLIF + cyst resection + neuronavigation	50% → 15%	7 → 2	0%	0	220	50	2	No	Solid fusion achieved	Marked improvement
Mean (Median)	55.3 (55)	—	—	—	—	60% → 19.3% (60% → 18%)	8.0 → 2.7 (8 → 2)	4.3% (3%)	1.0 (0)	213.3 (210)	66.7 (50)	2.3 (2)	0/3 (0%)	3/3 (100%) solid fusion	Clinical improvement in all patients

**Table 2:** Summary of clinical outcomes, surgical variables, and radiographic fusion status at 12-month follow-up. ODI:  Oswestry Disability Index; VAS: visual analog scale

Variable	Mean	Median
Age	55.3 years	55 years
ODI preoperative	60%	60%
ODI 1 month	19.30%	18%
ODI 12 months	4.30%	3%
VAS preoperative	8	8
VAS 1 month	2.7	2
VAS 12 months	1	0
Operative time	213.3 min	210 min
Estimated blood loss	66.7 mL	50 mL
Hospital stay	2.3 days	2 days
Perioperative complications	0/3 (0%)	—
Solid fusion at 12 months	3/3 (100%)	—

All patients presented with chronic low back pain associated with unilateral radiculopathy, paresthesias, and progressive functional limitation. MRI demonstrated L4-L5 LFSCs associated with degenerative facet arthropathy and segmental instability confirmed by dynamic radiographs. All patients underwent neural decompression, facet cyst resection, and navigation-assisted MIS-TLIF at L4-L5 with percutaneous pedicle screw placement guided by the O-arm system. No intraoperative or perioperative complications were observed.

At one-month follow-up, all patients demonstrated significant clinical improvement, including reduction of low back and radicular pain, improved functional status, and recovery of mobility. The mean ODI improved from 60% preoperatively to 19.3% postoperatively (median: 60% to 18%), while the mean VAS score improved from 8.0 to 2.7 (median: 8 to 2).

At the 12-month follow-up, sustained clinical improvement was observed in all patients. The mean ODI further improved to 4.3% (median: 3%), and the mean VAS score decreased to 1.0 (median: 0). Radiographic evaluation demonstrated solid fusion in all three patients (3/3, 100%), with stable instrumentation and no evidence of implant loosening, cage migration, or segmental instability.

The mean operative time was 213.3 minutes (median: 210 minutes), the mean estimated blood loss was 66.7 mL (median: 50 mL), and the mean length of hospital stay was 2.3 days (median: 2 days).

Histopathological examination confirmed LFSCs in all cases, demonstrating synovial lining with reactive synoviocyte proliferation and fibrocollagenous walls. In one case, a granulomatous reaction associated with multinucleated giant cells was identified.

## Discussion

LFSCs represent an advanced manifestation of the degenerative process of the lumbar spine and are closely associated with facet arthropathy, segmental hypermobility, and DS. Several studies have demonstrated that these lesions occur most frequently at the L4-L5 level, the most mobile lumbar segment, and are frequently associated with DS, supporting the concept of underlying segmental instability as a key pathogenic mechanism [1,9,10]. In our series, all patients presented with degenerative facet changes and associated spondylolisthesis, reinforcing the biomechanical theory of segmental instability as the main pathophysiological mechanism.

Currently, the literature considers facet synovial cysts not only as compressive lesions but also as radiological markers of lumbar degenerative instability. Saremi et al. (2024) described findings such as facet effusion, synovial cysts, and facet degeneration as part of the natural history of DS, progressing from a dysfunctional phase to instability and eventual restabilization [3].

Several radiological classifications have been proposed to guide the management of LFSCs, among which the NSURG classification is one of the most clinically applicable. The NSURG system integrates the degree of spinal canal compromise produced by the cyst with the degree of vertebral slip, using a 15% threshold to identify clinically relevant segmental instability. Within this system, grades 1-3 correspond to patients with vertebral slip <15% and increasing degrees of canal compromise, whereas grades 4 and 5 are characterized by vertebral slip ≥15%, reflecting clinically significant instability and a greater burden of neural compression. Campbell et al. demonstrated that grades 4 and 5 are associated with a higher risk of recurrence after decompression alone and may therefore benefit from concomitant fusion, whereas grades 1-3 generally demonstrate lower recurrence rates following decompression without stabilization [7].

Similarly, the anatomy-based classification proposed by Rosenstock and Vajkoczy categorizes LFSCs according to their anatomical location and associated spinal alignment abnormalities, providing guidance for the most appropriate surgical corridor while preserving facet integrity whenever possible. Importantly, cysts associated with spondylolisthesis or significant instability may benefit from concomitant segmental stabilization because of the increased risk of recurrence. These concepts are consistent with our treatment strategy, as all patients in our series presented with DS and underwent navigation-assisted MIS-TLIF, allowing both neural decompression and stabilization of the affected segment [4].

These findings are particularly relevant to our series, as they justify the use of navigation-assisted techniques and segmental stabilization in selected patients. Neuronavigation allowed targeted resection with less facet destruction, better bony preservation, and accurate anatomical orientation, especially in cases with severe degenerative anatomy. This approach may reduce the risk of postoperative instability progression described after extensive laminectomy or facetectomy procedures.

Historically, standard surgical treatment has consisted of open cyst resection through laminectomy or hemilaminectomy. Epstein (2004) reported that although decompression alone achieved significant clinical improvement, progression of DS occurred in a considerable proportion of patients and suggested considering primary fusion in cases associated with instability [10]. Similarly, Abd El-Kader (2016) observed favorable outcomes using decompression combined with transpedicular fixation and fusion, particularly in patients with concomitant DS [1].

In recent years, minimally invasive and endoscopic techniques have gained relevance because of their potential to preserve posterior stabilizing structures. Sukkarieh et al. (2015) demonstrated that minimally invasive resection through a contralateral tubular approach allowed adequate decompression with minimal blood loss, short hospital stay, and low morbidity [9].

Although surgery remains the most definitive treatment for symptomatic LFSCs, minimally invasive interventional strategies have emerged as potential alternatives in selected patients. Shah and Lutz (2003) reported high failure rates with aspiration and steroid injections, with approximately 50% of patients ultimately requiring surgical intervention [14]. More recently, Kim et al. (2022) and Park et al. (2023) described significant clinical improvement following percutaneous aspiration and rupture of facet synovial cysts, avoiding surgery in selected cases [13,16]. Similarly, radiofrequency-assisted techniques have been proposed as adjunctive minimally invasive options [17]. However, the available evidence remains limited to small series and case reports, and these approaches were not evaluated in the present study.

Despite these promising minimally invasive alternatives, the universal need for concomitant fusion remains controversial. Dantas et al. (2020) reported excellent functional outcomes using microsurgery without arthrodesis in a series of 50 patients, with a low rate of late reintervention, suggesting that decompression alone may be sufficient in carefully selected patients without significant instability [18]. Likewise, Terao et al. (2007) found adequate symptomatic resolution without concomitant fixation in most cases, although they described cyst recurrence and degenerative progression in some patients [5]. These findings suggest that the decision to perform fusion should be individualized according to the degree of instability, facet joint destruction, and the extent of bony resection required. Therefore, surgery with concomitant stabilization appears to remain preferable in patients with significant neural compression or associated segmental instability.

Another important aspect is the possibility of spontaneous regression, which has recently been documented in isolated cases. Kim et al. (2025) reported spontaneous regression of large LFSCs managed conservatively, emphasizing the importance of correlating clinical and imaging findings before indicating immediate surgery [15].

An additional consideration is the potential diagnostic challenge posed by LFSCs, as their radiological features may resemble those of other extradural spinal pathologies. Filali et al. (2024) emphasized the importance of correlating imaging findings with the clinical presentation to establish an accurate diagnosis and guide appropriate management [19].

Furthermore, Sarmiento et al. reported the development of an LFSC only one week after laminectomy in a patient with grade I DS and preoperative signs of instability, highlighting the potential role of persistent segmental instability in cyst formation and recurrence. The literature further suggests that up to one-third of patients with lumbar stenosis and stable DS may develop postoperative instability following decompression alone; therefore, concomitant fusion should be considered in selected cases to reduce recurrence and reoperation rates [20].

In our experience, the use of neuronavigation provided important advantages in terms of surgical precision, anatomical identification, and structural preservation, particularly in complex degenerative scenarios associated with spondylolisthesis. The combination of targeted decompression and selective stabilization allowed adequate clinical resolution with minimal tissue disruption while achieving solid radiographic fusion and sustained clinical improvement at 12-month follow-up in all patients. No cyst recurrence, implant failure, or revision surgery was observed during the 12-month follow-up period. Based on our experience, this approach appears to be a promising minimally invasive option for selected patients with LFSCs associated with degenerative instability; however, its potential advantages should be confirmed in larger comparative studies.

However, several limitations of the present study should be acknowledged. This report represents a retrospective case series of only three patients from a single institution and lacks a control group for comparison with decompression alone or other minimally invasive techniques. In addition, the retrospective design may introduce selection bias. Although all patients demonstrated favorable clinical outcomes and successful solid fusion at 12 months, the small sample size limits the generalizability of these results, and longer follow-up is necessary to evaluate long-term clinical and radiographic outcomes. Therefore, the present findings should be interpreted as descriptive observations that support the feasibility of this technique rather than evidence of its superiority over alternative surgical strategies.

Future research should focus on larger prospective multicenter studies with longer follow-up periods to better define the role of navigation-assisted MIS-TLIF in the management of LFSCs associated with degenerative instability. Comparative studies evaluating navigation-assisted MIS-TLIF against decompression alone, endoscopic procedures, and other minimally invasive approaches, as well as analyses of recurrence rates, patient-reported outcomes, cost-effectiveness, and radiographic fusion, will be essential to establish the optimal treatment strategy for this pathology.

## Conclusions

LFSCs associated with DS represent a manifestation of segmental lumbar instability in which both neural decompression and stabilization may be required. In this case series, navigation-assisted MIS-TLIF was associated with significant improvement in pain and functional disability, solid radiographic fusion at 12-month follow-up, and the absence of major perioperative complications or recurrence. These findings suggest that this technique is a feasible and safe surgical option for selected patients, allowing precise cyst resection while providing concomitant segmental stabilization through a minimally invasive approach. However, given the small sample size and the lack of a comparison group, these results should be interpreted as preliminary observations rather than evidence of superiority. Larger prospective multicenter studies with longer follow-up are needed to validate these findings and better define the role of navigation-assisted MIS-TLIF in the treatment of LFSCs associated with degenerative instability.

## References

[1] Abd El-Kader Hel-B (2016). Juxtafacet spinal synovial cysts. Asian Spine J.

[2] Francavilla TL, Weiss MC, Umansky D, Songhurst S, Davis RJ (2022). Patient outcomes after minimally invasive excision of lumbar synovial cysts with and without spondylolisthesis in an ambulatory care center setting. Int J Spine Surg.

[3] Saremi A, Goyal KK, Benzel EC, Orr RD (2024). Evolution of lumbar degenerative spondylolisthesis with key radiographic features. Spine J.

[4] Rosenstock T, Vajkoczy P (2020). New classification of facet joint synovial cysts. Acta Neurochir (Wien).

[5] Terao T, Takahashi H, Taniguchi M, Ide K, Shinozaki M, Nakauchi J, Kubota M (2007). Clinical characteristics and surgical management for juxtafacet cysts of the lumbar spine. Neurol Med Chir (Tokyo).

[6] Yoo YM, Kim KH (2024). Facet joint disorders: from diagnosis to treatment. Korean J Pain.

[7] Campbell R, Phan K, Mobbs R (2018). Classification of lumbar facet joint cysts using the NeuroSpine Surgery Research Group (NSURG) grading score and correlation with recurrence and clinical outcomes. World Neurosurg.

[8] Chaput C, Padon D, Rush J, Lenehan E, Rahm M (2007). The significance of increased fluid signal on magnetic resonance imaging in lumbar facets in relationship to degenerative spondylolisthesis. Spine (Phila Pa 1976).

[9] Sukkarieh HG, Hitchon PW, Awe O, Noeller J (2015). Minimally invasive resection of lumbar intraspinal synovial cysts via a contralateral approach: review of 13 cases. J Neurosurg Spine.

[10] Epstein NE (2004). Lumbar laminectomy for the resection of synovial cysts and coexisting lumbar spinal stenosis or degenerative spondylolisthesis: an outcome study. Spine (Phila Pa 1976).

[11] Liao S, Cheng L, Zhao Z, Zhu J, Huang F (2024). Distinct characteristics and progression patterns of facet joint structural lesions in radiographic axial spondyloarthritis. Ther Adv Musculoskelet Dis.

[12] Aggarwal A, Garg K (2021). Lumbar facet fluid: does it correlate with dynamic instability in degenerative spondylolisthesis? A systematic review and meta-analysis. World Neurosurg.

[13] Kim HT, Choi JH, Shin YD, Kim TY, Lee JY (2022). Successful treatment of symptomatic facet joint synovial cysts using C-arm fluoroscopy-guided percutaneous aspiration: report of two cases. Am J Transl Res.

[14] Shah RV, Lutz GE (2003). Lumbar intraspinal synovial cysts: conservative management and review of the world's literature. Spine J.

[15] Kim HJ, Ko JH, Chang DG (2025). Spontaneous regression of large-sized lumbar facet synovial cysts: two case reports and literature review. BMC Musculoskelet Disord.

[16] Park JW, Park JY, Seo HW, Shin YD, Park SH (2023). Successful treatment of facet joint synovial cyst through percutaneous rupture: a case report. Am J Transl Res.

[17] Hatgis J, Granville M, Berti A, Jacobson RE (2017). Targeted radiofrequency ablation as an adjunct in treatment of lumbar facet cysts. Cureus.

[18] Rolemberg Dantas FL, Dantas F, Vieira Caires AC, de Almeida Fonseca Filho G, Cariri GA, Botelho RV (2020). The results of microsurgery without fusion for lumbar synovial cysts: a case series of 50 patients. World Neurosurg.

[19] Filali Z, Briki A, Ferjani S, Aloui Y, Moulahi O, Haddad N (2024). A challenge diagnosis of lumbar synovial facet cyst. A case report. Int J Surg Case Rep.

[20] Sarmiento JM, Fourman MS, Lovecchio F, Lyons KW, Farmer JC (2022). Acute development of spinal lumbar synovial facet cyst within 1 week after lumbar decompression: illustrative case. J Neurosurg Case Lessons.

